# Barriers to biomedical care and use of traditional medicines for treatment of cervical cancer: an exploratory qualitative study in northern Uganda

**DOI:** 10.1111/ecc.12211

**Published:** 2014-06-13

**Authors:** A.D. Mwaka, E.S. Okello, C.G. Orach

**Affiliations:** Department of Medicine, School of Medicine, College of Health Sciences, Makerere University, Kampala; Department of Psychiatry, School of Medicine, College of Health Sciences, Makerere University, Kampala; Department of Community Health, School of Public Health, College of Health Sciences, Makerere University, Kampala, Uganda

**Keywords:** barriers to healthcare, traditional medicines, cervical cancer, northern Uganda

## Abstract

Use of traditional medicines for treatment of cancers has increased worldwide. We used a qualitative approach to explore barriers to biomedical care and reasons for use of traditional medicines for the treatment of cervical cancer in Gulu, northern Uganda. We carried out 24 focus group discussions involving men and women aged 18–59 years. We employed content analyses technique in data analysis. Traditional medicines were used mainly due to barriers to biomedical care for cervical cancer. The barriers included health system factors, for example long distances to health facilities and unavailability of medicines; health workers’ factors, for example negative attitudes towards patients and demands for bribes; individual patient’s factors, for example inability to pay for medical care; and socio-cultural beliefs about superiority of traditional medicines and perceived greater privacy in accessing traditional healers. Barriers to biomedical care and community beliefs in the effectiveness of traditional medicines encourage use of traditional medicines for treatment of cervical cancer but might hinder help-seeking at biomedical facilities. There is need for targeted culturally sensitive awareness campaign to promote effectiveness of modern medicine and to encourage cautious use of traditional medicines in the treatment of cervical cancer.

## Introduction

Traditional medicines or alternative therapies for cancer treatments are treatments other than conventional surgery, radiation and chemotherapy which are used concurrently with, before or after starting such conventional therapies (Cassileth 1996). Worldwide, the use of traditional medicines or alternative therapies has increased greatly in recent times especially among cancer patients (Pud *et al*. 2005; Scott *et al*. 2005; Tas *et al*. 2005; Eliott *et al*. 2008; Oh *et al*. 2010). In Australia, up to 61.5% of cancer patients used alternative and complementary medicines during their cancer treatment, and the independent predictors for use of alternative medicines included metastatic cancer, active religious practice and tertiary education (Klafke *et al*. 2012). In Turkey, the prevalence of traditional medicines use among cancer patients ranged from 54.5% to 61% (Ceylan *et al*. 2002; Nazik *et al*. 2012; Yildiz *et al*. 2013), while in Taiwan, the prevalence of traditional medicines use among advanced cancer patients was 64–79% (Liu *et al*. 1997; Ku & Koo 2012). In Nigeria, up to 65% of 160 cancer patients used traditional medicines (Ezeome & Anarado 2007). Some qualitative studies in South Africa and Ethiopia have reported lay people’s preferences for traditional medicines in the treatment of cervical cancer (Van Schalkwyk *et al*. 2008; Birhanu *et al*. 2012).

Concurrent use of traditional and or alternative and complementary medicines with modern medicines or recourse to traditional medicines as the main modality of treatment is common among patients with cancers (Ernst & Cassileth 1998; Sparber *et al*. 2000; Shen *et al*. 2002). Reasons for use of traditional medicines vary among cancer patients. Some cancer patients in the USA used alternative therapies to boost the immune system, improve the quality of life, prevent recurrence of cancer, and feel one is in control of their own lives (Nahleh & Tabbara 2003). In Malaysia, reasons for use of traditional medicines by breast cancer patients included recommendation by family members or friends, sanctioning by families, creditability of traditional healers, benefits of the traditional medicines over the modern medicines that were associated with delay in treatments and sometimes side-effects that patients needed to be cautious about and prevent (Muhamad *et al*. 2012). The use of traditional medicines was also associated with the search for therapies that are in line with traditional values, and beliefs and philosophy of life about health and illness (Astin 1998).

In sub-Saharan Africa, there is limited data on the rationale for and factors that predict use of traditional medicines by cervical cancer patients. This study sought to explore community views concerning reasons for use of traditional medicines for the treatment of cervical cancer, and barriers to quality biomedical care in northern Uganda in order to inform targeted interventions to increase access to biomedical facilities.

## Methods

### Study setting

This study was conducted in two counties (one rural, one urban) in Gulu district, northern Uganda. Gulu district and the surrounding region has two main hospitals in which diagnosis and surgical care for cervical cancer are performed; a private-not-for-profit and a public university teaching hospital, both of which are located in the municipality. The majority of the population in Gulu lived in internally displaced persons (IDP) camps for over 20 years due to a civil war. The IDP camps were only disbanded in 2006 (World Vision 2004), when people then moved back to their former villages where we interviewed them.

### Study design

We carried out a qualitative cross-sectional study using focus groups discussions (FGDs) to obtain shared experiences and views on barriers to seeking biomedical care and rationale for use or preference for traditional medicines for treatment of cervical cancer in the post conflict northern Uganda. We used FGDs because the technique generates a variety of information on an issue within a limited period (Rabiee 2004), and the social interactions between members of the FGDs can provide a window of opportunity to gain deeper insights into shared experiences and social norms (Krueger & Casey 2000) that would probably be obscured during a one-to-one interview (Richardson & Rabiee 2001).

### Sampling procedure

We purposefully selected two sites; a village in a rural county and another village in the municipality. In these two villages people had already resettled in their homesteads following the dismantlement of the IDP camps. In each village in Uganda, there is a committee of nine people elected by the members of the villages to represent and lead the villages. The chairpersons of these committees, the local council 1 (LC1) are gatekeepers to the communities and have lists of names of all the people in their villages. The LC1 chairpersons of the selected villages delivered personal invitation letters to purposefully sampled persons; invitation letters indicated the purpose, date, time and venues for the FGDs.

Eligible participants included; men and women aged 18–59 years who had lived in the study area for at least 18 years and knew the local language. We excluded participants with communication challenges and/or overt and documented mental illnesses, women with documented diagnosis of cervical cancer, men and women whose daughters had confirmed cervical cancer diagnoses, and men whose wives had confirmed cervical cancers. The participants directly affected by cervical cancer were excluded because their views might have been influenced during help-seeking for the disease.

### Data collection procedure

We collected data using pretested FGD guides. Discussions were facilitated by experienced research assistants with bachelor degrees in social sciences and education. The research assistants were trained for 2 days. They also participated in the translation of the study tools into the local language and they piloted the tools prior to data collection. The first author participated in eight of the 24 FGDs. We collected data between May and June 2012. The proceedings of all the FGDs were documented by a note taker. Digital audio-recordings were done to preserve details of discussions. We conducted separate FGDs for men and women, categorised into the following age groups: 18–29 years; 30–34 years; 35–44 years; and 45–59 years respectively (Table 1). Each FGD had 10–12 members.

### Data analysis

Field notes and recorded information were transcribed and translated into English. We used content analysis (Graneheim & Lundman 2004; Silverman 2011) approach to analyse data. The first author read through all the transcripts while listening to the audio recordings to clarify data source, get familiarised with the data and confirm completeness of transcription and accuracy of translation. Then two of the authors, including the first author read six transcripts and independently developed codes relating to the sections on barriers to seeking biomedical care and use of traditional medicines for treatment of cervical cancer. The authors then met, discussed and agreed on codes and code definitions to be used in coding the data. The first author then coded all the data. We used the software, ATLAS.ti Version 6.1 in coding, tracking changes and organising data during analysis. We used the codes to retrieve various segments of the data, wrote memos and developed final themes and categories from data. We used typical verbatim quotes from participants to illustrate the key issues in the themes and categories. Interpretations were done with considerations to gender, age groups and whether the participants lived in the rural or urban setting.

### Ethical approval and consent

Approval for this study was provided by the Makerere University School of Medicine Ethics Review Committee (SOMREC) and the Uganda National Council of Science and Technology (UNCST). Permission to access the communities were obtained from Gulu district local leaders including the LC1 chairpersons of the study villages. We explained the purpose of the study to participants of the FGDs before starting discussions. The participants provided oral informed consents and signed or applied a thumb print to register to participate in the study. Additional verbal permissions were obtained from participants to allow audio-recording of the discussions. A uniform transport refund was provided to the study participants.

## Results

Barriers to biomedical care for cervical cancer included: health systems related barriers, health workers’ related barriers, individual patients’ characteristics and sociocultural beliefs related to the convenience, ready availability, low cost and high efficacy of traditional medicines. The results are presented along with typical quotes, identified by the study site (urban/rural), gender and age group. Letters ‘A’ and ‘B’ were used to show the order in which different FGDs of same age, gender and site categories were reported in this paper, for example (Urban; men, aged 18–29 years, A, and Urban; men, aged 18–29 years, B).

### Health system related barriers

Health system-related barriers including long distances to the facilities, high cost of transport to health facilities, congestions and long waiting times for consultation and treatment, lengthy procedures, and frequent unavailability of medicines were reported to hinder help-seeking at biomedical facilities.

#### Long distances to health facilities

The majority of participants reported that most public health facilities are located far away and people travelled long distances to access services, whereas traditional practitioners were within the communities and easy to access.

The long distances to health centre for some people compared with the traditional healers who are readily available encourages them to use traditional medicines. (Urban; men, aged 18–29 years, A)

#### High cost of transport to biomedical facilities and unaffordable medical bills

Most people depended on public transport to reach health units. Lack of transport money was repeatedly mentioned as a key reason for resorting to the use of traditional medicines. This challenge was expressed more in the rural FGDs in comparison with the urban FGDs, where concerns were more on the cost of transport to the city in case of referral for more specialised care in Mulago national referral hospital, the only hospital in Uganda where radiotherapy services are provided.

Another problem is the difficulty in accessing transport especially the high cost of transport to town and also outside of Gulu in case you are referred to Mulago and also you fear that you will get lost. (Urban; men, aged 30–44 years, A)

In both the rural and urban FGDs, participants were concerned with the unaffordable cost of healthcare in modern health units.

There is no money to pay for transport and medical bills. They (health professionals) ask you to buy gloves, razor blades, carpet; and when you do not have, they chase you away. (Urban; women, aged 18–29 years, A)

#### Congestions and long waiting times for consultations and treatment

In five FGDs, participants were concerned with the congestions at the health facilities, long waiting times to consult the health workers, leading to a waste of patients’ valuable time for other important activities such as gardening.

Health workers delay to respond to you and you spend a lot of time there. One would rather go to the traditional healer because there are too many people in hospitals where you take a lot of time. (Urban; men, aged 30–44 years, A)

Participants in the majority of FGDs gave testimonies of circumstances when their conditions got worse while waiting to be helped in hospitals. Some participants reported their own experiences with healthcare system while seeking help for other conditions. For example, some women reported going into labour and delivering at the health units without the expert assistance of the professionals.

I have my personal example when a medical personnel left me in the ward and said he was still going home; he came back when I had given birth. (Urban; women, aged 18–29 years, A)

#### Lengthy, unclear and unfamiliar processes in hospitals

In four FGDs, the participants expressed disappointment with the hospital bureaucracy whereby patients were told to move from one section to another and/or had to return another day for further investigations before medicines for their illnesses were dispensed. For the participants, when a patient goes to the hospital, it is important that medicines are dispensed immediately.

From the hospital, they keep tossing you around, telling you to come tomorrow until you just give up … such a person may not come back to the hospital again. (Urban; men, aged 45–59 years, B)

The participants from the rural FGDs were concerned with inadequate guidance within health facilities. Participants acknowledged the frustration patients go through when they get stranded within the hospitals as they navigate from one section of the facility to another. It was concerning to participants that health professionals do not provide proper directions and hospital units were either not labelled or labelled in English, a language not understood by many people from the villages.

From the hospital, most people do not know where to go next especially if you cannot read the signs and directions; and there is usually nobody to give these directions forcing people not to go back to the hospital. (Rural; women, aged 45–59 years, A)

#### Medicines unavailability and pilfering by health workers

Participants in the majority of FGDs, both rural and urban reported that, frequent unavailability of medicines in the public health units often led patients to be directed by the health workers to buy the medicines from elsewhere.

Many times there are no medicines in the health centre and they instead refer you to go and buy from their clinics; this makes some people not to go to health facilities at all because they know they will come back home empty handed. (Rural; men, aged 45–59 years, A)

The complaints about the unavailability of medicines cut across all age groups from both rural and urban areas.

Some of the health centres only have the buildings but there are no medicines inside; people feel that it is a waste of time to go there and be told that there is no medicine in the health centre. (Rural; women, aged 18–29 years, A)

Sometimes there is no medicine in the health centre so instead of wasting time going there, you just sit back and look for traditional medicines. (Urban; men, aged 45–59 years, B)

The challenge of medicines’ unavailability was thought to result from the health professionals stealing medicines from public hospitals.

The health workers take the medicine to their clinics and when you go to the hospital, they tell you there is no medicine but refer you to their clinics. (Urban; women, aged 18–29 years, A)

### Health provider related barriers

The participants said that women sometimes resort to traditional medicines for treatment of cervical cancer because some health workers discriminate, disrespect, demand bribes and use languages that are not understood by the patients and their families.

#### Discriminations of patients by health workers

There was general consensus that many health professionals despised patients and differentially treated patients; with the rich and neatly dressed people attended to first, irrespective of their position in the queue.

Health workers have this habit of classifying people according to education levels. They respond faster to people who dress well and seem to have money leaving us the village folks last. They look at your luggage and if you use a ‘kavera’ (plastic bags) to carry your belongings, you will be last in the queue. (Rural; women, aged 18–29 years, B)

Complaints about differential treatment were common also among the urban FGD participants.

Health workers also show favours to people they know and people who appear to have money or educated and leave us the poor ones last. (Urban; men, aged 30–44 years, A)

#### Disrespect by health professionals

Participants in half of the 24 FGDs reported that some people avoided the health units and preferred traditional medicines because of the arrogance, disrespect and undesirable comments from some of the health professionals in the modern health units.

Some of the health workers are so arrogant and say very bad things to the patients that the patients actually fear going to the health centres. For example there was this one who told an old man that he is wasting medicines meant for young people. (Rural; women, aged 18–29 years, A)

Participants, particularly the men from rural FGDs also reported frequent use of abusive language by health professionals.

Arrogance and use of abusive language from the medical workers on the patients and their caregivers makes people lose interest in the health centres given that no one would like to be insulted all the time. (Rural; men, aged 18–29 years, A)

#### Demands for bribes

Some health professionals reportedly demanded unofficial payments in the form of bribes before they offered services to the patients. Patients who cannot afford such payments were reportedly served last or turned away. The concerns about bribes were reported more from the urban FGDs (four) than the rural FGDs.

Some people pay bribes to doctors who treat them first and they ignore you the poor one or ask for bribe from you also. They also segregate according to status and money, making us the poor ones feel rejected and promise never to go there again. (Urban; men, aged 18–29 years, A)

The older women from the urban FGDs also said that demands for bribes were common in the health units and the amounts of money asked for were sometimes exorbitant.

They also ask for too much money from you before they give you treatment in form of bribe or motivation to make them work. (Urban; women, aged 45–59 years, A)

#### Language barrier

The health workers, including those who know the local language often preferred to talk in English, despite many rural people especially elderly women not understanding English. This view was more common in the rural FGDs.

The health workers speak only in English and for some of us who cannot speak the language we get lost and fear going back again. (Rural; women, aged 45–59 years, A)

The men from the rural FGDs also had similar concerns about language barrier;

Some members of the community fear communicating in English and would rather not go to the hospitals at all because they will not communicate. (Rural; men, aged 18–29 years, B)

### Socio-cultural beliefs in traditional medicines

Participants discussed several reasons for continued use of traditional medicines in the region. These included cheaply available and effective traditional medicines, competing demands for women’s time, influence of traditional healers, fear of embarrassment and stigma when diagnosed with certain diseases, and fear of diagnosis of serious diseases.

#### Traditional medicines are available, cheaper and payment system more convenient

In a quarter of the FGDs, the participants were concerned with the intrinsic benefits derived from traditional medicines, which makes their use more desirable and preferred in the communities. In addition, traditional medicines were considered cheaper and could be paid for by instalments.

Some people prefer traditional healers to health centres because traditional medicine is cheaper; you can take treatment and pay later or until you are cured. (Rural; women, aged 18–29 years, B)

Respondents reported no single best treatment for cervical cancer and that trying different medicines made treatment of the disease very expensive for patients; thus making cheaper treatment options more preferable.

The person also spends a lot of money on treatment since there is no clear cut treatment for the disease considering that the person has to keep going to the health centre all the time and also try other treatments like traditional medicines and yet because of the illness the patient cannot work hard enough to earn money for treatment. (Rural; men, aged 45–59 years, A)

#### Competing demands for time and resources

In both the rural and urban FGDs, participants reported that people sometimes did not go to biomedical facilities because of competing commitments. There were concerns with in-hospital admissions when seeking help at bio-medical facilities. This would conflict with other roles including food production activities.

Some people fear that when they go to the health centre, they will be admitted and therefore spend long in the health centre leaving their crops in the field which will get destroyed. They prefer to attend to their crops first. (Rural; men, aged 45–59 years, A)

Most urban participants reiterated similar ideas for the lack of time for consultations at biomedical facilities.

People normally first want to finish the businesses they have at hand before going to the hospital. For example one would first want to finish weeding the garden before going to the hospital. (Urban; men, aged 45–59 years, B)

#### Perceived incurability of cancers and influence of traditional healers

Traditional healers deliberately promoted the use of traditional medicines by convincing people that traditional medicines cured cancers. In two FGDs, most of the participants expressed the belief that modern medicines did not cure cervical cancer.

Because there is no treatment for cancer in health centres, the traditional healers and diviners deceive people that they have the cure for cancers, so that patients go to their traditional practices. (Urban; men, aged 18–29 years, B)

Belief in the effectiveness of traditional medicines on cervical cancer was ubiquitous in both rural and urban sites. Participants referred to circumstances when traditional medicines led to the cure of cervical cancer. The evidences provided to illustrate the effectiveness of traditional medicines included a case of a 16-year-old girl who actually may have not had cervical cancer but some other bleeding disorder.

In my opinion, I think the medicine especially the ones given from the health centre only reduces the severity of the disease but cannot cure it. It is the traditional medicines that can cure this disease. We have also seen an example of a 16-year-old girl who got cured using traditional medicine. She was brought from Kampala. (Rural; women, aged 45–59 years, A)

To the extent that modern medicine is believed to be ineffective in the cure of cervical cancer, people would not find any reason to go to, or recommend the use of biomedical facilities if they believed or knew they had cervical cancer.

Some people actually think the disease (cervical cancer) cannot be cured so it makes no sense to waste the little resources they have on medical bills. They prefer to explore cheaper options like traditional medicines or simply staying at home. (Rural; women, aged 18–29 years, A)

#### Fear of embarrassment

In more than a third of FGDs, most participants reported that traditional medicines were preferred by people because, unlike in the hospitals, there was privacy and confidentiality in traditional medical practices.

There is also a lot of embarrassment on the patients because of having blood stained dress, the bad smell and fear that the flow will start in public which may prevent the patients from going to the health centre or to appear in public. (Rural; women, aged 45–59 years, A)

Patients with diseases associated with sexual transmission or other forbidden actions get embarrassed and might be stigmatised in the community when other people get to know they have such illnesses.

Some people fear going to the hospital because the disease is embarrassing like syphilis; so people prefer traditional treatment since not many people will know about it if treated traditionally. (Rural; women, aged 18–29 years, B)

#### Fear of diagnosis of a serious disease

Some participants from the urban FGDs said that people sometimes avoided biomedical facilities for symptoms of their diseases because of the fear of being investigated and finding out that they have a disease that cannot easily be cured or cannot be cured at all, for example cancers.

Some people fear that doctors will uncover diseases that will not cure or difficult to treat and this may upset them or cause a lot of worries. (Urban; women, aged 18–29 years, B)

## Discussions

Lay people use traditional medicines for treatment of cancers and other illnesses mainly because of the myriad of barriers to accessing services in biomedical facilities. A key barrier to help-seeking was the long distances to the few health facilities where cervical cancer can be diagnosed and managed. The costs of transportation to the health facilities were considered prohibitive to help-seeking. Travelling the long distances on foot, bicycles, motorcycles or public vehicles, chronic shortages of health professionals and the unavailability of medicines were experienced at many health facilities. In addition, the multiple referrals from the lower level health units which are a lot nearer to people’s residences to the better equipped hospitals further away in Gulu town, require several visits at each stage, plus the spending of lots of time and money in consultations and travels. Even when patients managed to reach health facilities, long waiting times at the facilities was also considered a major hindrance to the use of biomedical services. Many patients and/or the limited number of health professionals and skills mix might account in part for the long time spent waiting to consult and receive professional services of the health workers. Therefore, instead of travelling long distances and ending up at health facilities that do not deliver the expected services, women sometimes resorted to traditional treatment. In Uganda, similar findings have been reported by other investigators assessing reasons for use of traditional medicines by diabetic patients (Rutebemberwa *et al*. 2013) and tuberculosis patients (Buregyeya *et al*. 2011). In Malawi long distances to health facilities and the lack of transportation were found to be the key reasons for the delay for help-seeking by cervical cancer patients. Other reasons were the dynamics of care, punctuated with several visits and referral from one level to another (Chadza *et al*. 2012). In a study from Tanzania, it was found that long distances coupled with the lack of transportation and poor roads hindered access to health facilities (Kunda *et al*. 2007).

Accessibility to cervical cancer care was viewed at two levels; access to the local health facilities and access to the specialised facilities for cancer services. Some participants expressed great disappointments with the Uganda health system which has failed to make specialised treatments such as *‘burning’* (referring to radiotherapy and chemotherapy) for cervical cancer available and accessible to people who need it. Distance to cervical cancer treatment points often made the journey to these points impossible because of financial constraints, and/or lack of relatives in the city to guide them. Radiotherapy, a central modality of treatment for late stage cervical cancer is scarce in sub-Saharan Africa. Barton and colleagues found that the supply of radiation machines was inadequate to meet demand (Barton *et al*. 2006). Similarly, surgical and other specialised cervical cancer care are equally scarce in most of sub-Saharan Africa (Kingham *et al*. 2013). It is important to note that for the poor, travel costs may exhaust any financial reserves before the first cancer treatment is offered.

With public health facilities often lacking medicines and medical bills at the private facilities considered prohibitive, people would seek alternative care in traditional medicine practices. Lack of finances for healthcare and transport was found to be a barrier to quality biomedical care among the poor of Tanzania (Mamdani & Bangser 2004) and among Hispanics in the USA (Van Oss Marin *et al*. 1983). High costs of transportation, medicines and medical tests might also hamper access to biomedical care and hence see people resort to traditional alternative care.

Use of the English language by health workers at biomedical facilities was another barrier that encouraged women and other people to seek care with the traditional healers where they are able to explain themselves eloquently in the local language. Being understood seems an important element in the choice of source of help-seeking in this study community. In the USA, a review of 47 studies showed that language barrier was associated with less frequent clinic visits and less satisfaction with services among other inconveniences (Yeo 2004).

Labelling of service points in the health facilities is often done in English and may not provide the necessary guidance for patients to get to the next service points. This was especially bound to be the case for the majority population of older women who do not understand English. Consequently, patients may get lost within the facilities, waste a lot of time and sometimes get so frustrated that they go back home without having received any care. In such cases, participants might seek other means of care such as traditional medicines to avoid repeated frustrations. Labelling the various units and departments and having pictorial posters, for example a picture of a needle and syringe to show injection rooms might go a long way to minimising the frustrations in patients in finding their next destinations within the health facilities. It might be important to write directions in both English and the local languages.

Health professionals’ absenteeism and long waiting times at health facilities were reported in five FGDs as a barrier to help-seeking. Health professionals are reported to sometimes leave their duty stations early and move on to other jobs or their private practices in order to make ends meet. To improve their own livelihoods, doctors and other health professionals find themselves engaging in a second job, commonly referred to as moonlighting, and/or operating private clinics, both of which make doctors less available at the public health facilities (Roenen *et al*. 1997).

Frequent lack of medicines in the public health facilities was a common phenomenon that made help-seeking in the health facilities meaningless. Some participants were distraught that health workers refer patients to their personal clinics to buy the same medicines allegedly stolen from the health facilities where the medicines should normally be provided to them free of charge. In Mozambique and Cape Verde, a study revealed that health professionals inevitably engaged in some forms of misuse of their privileged position of access to pharmaceuticals and other hospital resources to aid their own survival (Ferrinho *et al*. 2004). While governments and other stakeholders work tirelessly to deal with the problem of the unavailability of medicines and pilfering, the communities in Uganda need to be encouraged to continue to seek care in healthcare facilities so that the impact of disease on individuals and communities might be minimised.

The participants in this study were concerned about negative attitudes of some health workers and demands for bribes before delivering services at public health facilities. It is reported that health workers sometimes directly or indirectly demand bribes which the poor cannot afford to pay. Participants also claimed that health workers selectively offer services to the wealthy, educated and well dressed patients before serving the very poor patients even if the latter had queued much earlier. In effect, participants felt discriminated against and neglected at the public health facilities. In addition, some participants reported that health workers often act arrogantly to patients. Thus, some people reportedly avoid the alleged inhumane treatment, demands for bribes and discrimination by resorting to traditional healers where they are attended to promptly and without discrimination of any kind. Demands for unofficial payments and corruption at health facilities have been reported in other studies. A study among public health doctors revealed that receiving gifts from patients is one of the ways for doctors to survive (Roenen *et al*. 1997). Whether these gifts are solicited or not, it is possible that patients themselves may consider such gestures as indirect bribery for services at the time or in future help-seeking. Keeping health professionals motivated in their work, for example through improvement in remunerations, acknowledgement of their efforts by community leaders, and providing welfare schemes to cater for the education and healthcare of their families may reduce the health professionals’ alleged demands for bribes.

Another barrier to help-seeking that might promote the use of traditional medicines is the multiple demands on women’s time and resources. Women in the study community and most of sub-Saharan Africa do most of the domestic and gardening work; studies show that in Africa 70–80% of agricultural production is done by women (Ogunlela & Mukhtar 2009). When women get sick during the farming season, they might resort to the use of traditional medicines until they can get time off from their gardening and household chores. It was found that caretakers’ other responsibilities in the home are key barriers to the continuity of care, for example, in the case of children with clubfoot in Uganda (McElroy *et al*. 2007). Women need to be educated on the need to prioritise their health in relation to their gardening and other responsibilities in the homes, so that better health might enable them to be more productive. It is also important for governments and other stakeholders in health to make health services more accessible and user friendly to the communities.

In this study, the majority of participants revealed that the use of traditional medicines did not occur simply as a response to the inadequacy of the modern health system but also because the traditional medicines, over generations have shown to be effective and useful in treating several illnesses. This means that irrespective of the barriers discussed earlier, people in this region would still seek care from practitioners in traditional medicine. Traditional medicines are being used in many countries for different illnesses. In Uganda, recent studies showed that diabetic (Rutebemberwa *et al*. 2013) and tuberculosis (Buregyeya *et al*. 2011) patients believed strongly in the intrinsic efficacy and effectiveness of traditional medicines for their ailments. In Turkey, the majority of cancer patients have been reported to have used traditional medicines at some point in their cancer journey (Akyol & Oz 2011). Most of the patients in the Turkish study reported that they obtained information about the use of herbal remedies from close friends, family members or the media. The fact that community members influence the choices of care by cancer patients underscores the need to explore the community beliefs that establish the rationale for the use of traditional medicines. The use of complementary and alternative medicine around the world has increased dramatically in recent times and its use by patients living with cancer is particularly common (Ernst 2003; Pud *et al*. 2005; Scott *et al*. 2005; Tas *et al*. 2005; Eliott *et al*. 2008).

Participants in this study reported that people go to traditional healers particularly when they have certain diseases that they do not wish other people to know about, for example sexually transmitted diseases. The participants felt that the privacy in public health facilities was not good and that other people may overhear their diagnoses when they are consulting with the health providers. Other studies have shown that people with sexually transmitted infections prefer traditional healers and only go to modern medicine later (Zachariah *et al*. 2002).

Fear of a bad diagnosis from biomedical facilities, for example cancer, was reported as a reason for using traditional medicines, where treatment is administered on the basis of symptoms rather than a firm diagnosis. People who fear their symptoms might be due to some severe diseases that may not be curable would prefer to simply treat the disease without being told the name. Fear of a cancer diagnosis was found to be a barrier for seeking screening for cervical cancer in Latin America (Agurto *et al*. 2004). Demystifying cancers through targeted health education programmes may mitigate the fear of a cancer diagnosis and encourage help-seeking at biomedical facilities where adequate and timely care maybe provided.

### Limitations

Transferability of our findings to other parts of Uganda as well as to other low income countries needs to take into account the geopolitical and socioeconomic circumstances of northern Uganda as a region that experienced civil conflict continuously for more than 26 years and as one of the poorest regions within Uganda. The trust of the war afflicted people in modern medicine, the destabilisation of the cultural set up of the people and the high level of poverty in the region could individually or in combination have influenced people’s help-seeking and preferred practices. Second, the accounts provided by the participants of this study on barriers to biomedical care and rationale for use of traditional medicines might be partial, and may be dependent on the lived circumstances of the participants.

### Strengths of this study

The use of focus group discussions with homogenous groups could have allowed for free expressions among the group participants and therefore freedom to provide detailed personal and observed experiences on the discourse of this study. Qualitative data collection methods such as FGDs lend themselves to detailed explorations of issues such as beliefs and opinions on the rationale for use of traditional medicines and barriers experienced in seeking care at biomedical facilities for cervical cancer and other disorders. Content analysis with verbatim quotations of participants has added validity and trustworthiness to the accounts in this study.

## Conclusions

Use of traditional medicines is promoted by two main factors: (1) the multitude of barriers to access of biomedical facilities such as the unavailability of medicines, unaffordable medical bills, the cost of transport to facilities, long waiting times at facilities, absenteeism of health workers, discrimination and disrespect by some health workers, fear of embarrassment when diagnosed with some diseases, and language barriers; and (2) the beliefs about the intrinsic efficacy and effectiveness of traditional medicines for many illnesses, greater confidentiality afforded at traditional medicine practices because of the fewer people there at any one time as compared with modern medical practices where there are several people, and the cheap cost and convenient methods of payments for traditional medicines.

Our research findings suggest the need to strengthen public health education programmes to promote the use of biomedical facilities for cervical cancer so that the health of women and their productivity might be improved. There is a need to address the allegations of bribery, absenteeism and disrespectful behaviours by the health professionals in order to encourage the population to regain trust in the health system and increase utilisation of the biomedical facilities in the region.

## Figures and Tables

**Table 1:** Distribution of focus groups discussions by residence, gender and age groups


	Residence of participants				
Age group (years)	Rural		Urban		
	Men	Women	Men	Women	Total

18–29	2	2	2	2	8
30–44	1	2	3	1	7
45–59	3	2	3	1	9
Total	6	6	8	4	24


## References

[R1] Agurto I, Bishop A, Sanchez G, Betancourt Z, Robles S (2004). Perceived barriers and benefits to cervical cancer screening in Latin America. Preventive Medicine.

[R2] Akyol AD, Oz B (2011). The use of complementary and alternative medicine by patients with cancer: in Turkey. Complementary Therapies in Clinical Practice.

[R3] Astin JA (1998). Why patients use alternative medicine: results of a national study. JAMA: The Journal of the American Medical Association.

[R4] Barton MB, Frommer M, Shafiq J (2006). Role of radiotherapy in cancer control in low-income and middle-income countries. The Lancet Oncology.

[R5] Birhanu Z, Abdissa A, Belachew T, Deribew A, Segni H, Tsu V, Mulholland K, Russell FM (2012). Health seeking behavior for cervical cancer in Ethiopia: a qualitative study. International Journal for Equity in Health.

[R6] Buregyeya E, Kulane A, Colebunders R, Wajja A, Kiguli J, Mayanja H, Musoke P, Pariyo G, Mitchell EM (2011). Tuberculosis knowledge, attitudes and health-seeking behaviour in rural Uganda. The International Journal of Tuberculosis and Lung Disease.

[R7] Cassileth BR (1996). Alternative and complementary cancer treatments. The Oncologist.

[R8] Ceylan S, Hamzaoglu O, Komurcu S, Beyan C, Yalcin A (2002). Survey of the use of complementary and alternative medicine among Turkish cancer patients. Complementary Therapies in Medicine.

[R9] Chadza E, Chirwa E, Maluwa A, Malata A, Kazembe A, Chimwaza A (2012). Factors that contribute to delay in seeking cervical cancer diagnosis and treatment among women in Malawi. Health.

[R10] Eliott JA, Kealey CP, Olver IN (2008). (Using) complementary and alternative medicine: the perceptions of palliative patients with cancer. Journal of Palliative Medicine.

[R11] Ernst E (2003). The current position of complementary/alternative medicine in cancer. European Journal of Cancer.

[R12] Ernst E, Cassileth BR (1998). The prevalence of complementary/alternative medicine in cancer: a systematic review. Cancer.

[R13] Ezeome ER, Anarado AN (2007). Use of complementary and alternative medicine by cancer patients at the University of Nigeria Teaching Hospital, Enugu, Nigeria. BMC Complementary and Alternative Medicine.

[R14] Ferrinho P, Omar MC, Fernandes MD, Blaise P, Bugalho AM, Lerberghe WV (2004). Pilfering for survival: how health workers use access to drugs as a coping strategy. Human Resources for Health.

[R15] Graneheim UH, Lundman B (2004). Qualitative content analysis in nursing research: concepts, procedures and measures to achieve trustworthiness. Nurse Education Today.

[R16] Kingham TP, Alatise OI, Vanderpuye V, Casper C, Abantanga FA, Kamara TB, Olopade OI, Habeebu M, Abdulkareem FB, Denny L (2013). Treatment of cancer in sub-Saharan Africa. The Lancet Oncology.

[R17] Klafke N, Eliott JA, Wittert GA, Olver IN (2012). Prevalence and predictors of complementary and alternative medicine (CAM) use by men in Australian cancer outpatient services. Annals of Oncology.

[R18] Krueger RA, Casey MA (2000). Focus Groups: A Practical Guide for Applied Research.

[R19] Ku CF, Koo M (2012). Association of distress symptoms and use of complementary medicine among patients with cancer. Journal of Clinical Nursing.

[R20] Kunda J, Fitzpatrick J, Kazwala R, French NP, Shirima G, Macmillan A, Kambarage D, Bronsvoort M, Cleaveland S (2007). Health-seeking behaviour of human brucellosis cases in rural Tanzania. BMC Public Health.

[R21] Liu JM, Chu HC, Chin YH, Chen YM, Hsieh RK, Chiou TJ, Whang-Peng J (1997). Cross sectional study of use of alternative medicines in Chinese cancer patients. Japanese Journal of Clinical Oncology.

[R22] Mamdani M, Bangser M (2004). Poor people’s experiences of health services in Tanzania: a literature review. Reproductive Health Matters.

[R23] McElroy T, Konde-Lule J, Neema S, Gitta S (2007). Understanding the barriers to clubfoot treatment adherence in Uganda: a rapid ethnographic study. Disability and Rehabilitation.

[R24] Muhamad M, Merriam S, Suhami N (2012). Why breast cancer patients seek traditional healers. International Journal of Breast Cancer.

[R25] Nahleh Z, Tabbara IA (2003). Complementary and alternative medicine in breast cancer patients. Palliative and Supportive Care.

[R26] Nazik E, Nazik H, Api M, Kale A, Aksu M (2012). Complementary and alternative medicine use by gynecologic oncology patients in Turkey. Asian Pacific Journal of Cancer Prevention.

[R27] Ogunlela IY, Mukhtar AA (2009). Gender issues in agriculture and rural development in Nigeria: the role of women. Humanity and Social Sciences Journal.

[R28] Oh B, Butow P, Mullan B, Beale P, Pavlakis N, Rosenthal D, Clarke S (2010). The use and perceived benefits resulting from the use of complementary and alternative medicine by cancer patients in Australia. Asia-Pacific Journal of Clinical Oncology.

[R29] Pud D, Kaner E, Morag A, Ben-Ami S, Yaffe A (2005). Use of complementary and alternative medicine among cancer patients in Israel. European Journal of Oncology Nursing.

[R30] Rabiee F (2004). Focus-group interview and data analysis. The Proceedings of the Nutrition Society.

[R31] Richardson CA, Rabiee F (2001). A question of access: an exploration of the factors that influence the health of young males aged 15 to 19 living in Corby and their use of health care services. Health Education Journal.

[R32] Roenen C, Ferrinho P, Van Dormael M, Conceicao MC, Van Lerberghe W (1997). How African doctors make ends meet: an exploration. Tropical Medicine and International Health.

[R33] Rutebemberwa E, Lubega M, Katureebe SK, Oundo A, Kiweewa F, Mukanga D (2013). Use of traditional medicine for the treatment of diabetes in Eastern Uganda: a qualitative exploration of reasons for choice. BMC International Health and Human Rights.

[R34] Scott JA, Kearney N, Hummerston S, Molassiotis A (2005). Use of complementary and alternative medicine in patients with cancer: a UK survey. European Journal of Oncology Nursing.

[R35] Shen J, Andersen R, Albert PS, Wenger N, Glaspy J, Cole M, Shekelle P (2002). Use of complementary/alternative therapies by women with advanced-stage breast cancer. BMC Complementary and Alternative Medicine.

[R36] Silverman D (2011). Interpreting Qualitative Data. A Guide to the Principles of Qualitative Research.

[R37] Sparber A, Bauer L, Curt G, Eisenberg D, Levin T, Parks S, Steinberg SM, Wootton J (2000). Use of complementary medicine by adult patients participating in cancer clinical trials. Oncology Nursing Forum.

[R38] Tas F, Ustuner Z, Can G, Eralp Y, Camlica H, Basaran M, Karagol H, Sakar B, Disci R, Topuz E (2005). The prevalence and determinants of the use of complementary and alternative medicine in adult Turkish cancer patients. Acta Oncologica (Stockholm, Sweden).

[R39] Van Oss Marin B, Marin G, Padilla MA, De La Rocha C (1983). Utilization of traditional and non-traditional sources of health care among Hispanics. Hispanic Journal of Behavioral Sciences.

[R40] Van Schalkwyk SL, Maree JE, Wright SC (2008). Cervical cancer: the route from signs and symptoms to treatment in South Africa. Reproductive Health Matters.

[R41] World Vision (2004). World Vision, Pawns of Politics: Children, Conflict and Peace in Northern Uganda.

[R42] Yeo S (2004). Language barriers and access to care. Annual Review of Nursing Research.

[R43] Yildiz I, Ozguroglu M, Toptas T, Turna H, Sen F, Yildiz M (2013). Patterns of complementary and alternative medicine use among Turkish cancer patients. Journal of Palliative Medicine.

[R44] Zachariah R, Nkhoma W, Harries AD, Arendt V, Chantulo A, Spielmann MP, Mbereko MP, Buhendwa L (2002). Health seeking and sexual behaviour in patients with sexually transmitted infections: the importance of traditional healers in Thyolo, Malawi. Sexually Transmitted Infections.

